# Sofosbuvir and ribavirin before liver re-transplantation for graft failure due to recurrent hepatitis C: a case report

**DOI:** 10.1186/s12876-015-0259-5

**Published:** 2015-03-26

**Authors:** Julien Vionnet, Manuel Pascual, Haithem Chtioui, Emiliano Giostra, Pietro E Majno, Laurent A Decosterd, Darius Moradpour

**Affiliations:** 1 Service of Gastroenterology and Hepatology, Centre Hospitalier Universitaire Vaudois, University of Lausanne, Lausanne, Switzerland; 2 Transplantation Center, Centre Hospitalier Universitaire Vaudois, University of Lausanne, Lausanne, Switzerland; 3 Service of Immunology and Allergy, Centre Hospitalier Universitaire Vaudois, University of Lausanne, Lausanne, Switzerland; 4 Laboratory and Division of Clinical Pharmacology, Service of Biomedicine, Centre Hospitalier Universitaire Vaudois, University of Lausanne, Lausanne, Switzerland; 5 Service of Gastroenterology and Hepatology, Geneva University Hospitals, Geneva, Switzerland; 6 Services of Transplant and Abdominal Surgery, Geneva University Hospitals, Geneva, Switzerland

**Keywords:** Directly acting antiviral, Hepatitis C virus, Liver transplantation, Therapeutic drug monitoring

## Abstract

**Background:**

Recurrent hepatitis C virus infection after liver transplantation is associated with reduced graft and patient survival. Re-transplantation for graft failure due to recurrent hepatitis C is controversial and not performed in all centers.

**Case presentation:**

We describe a 54-year-old patient with hepatitis C virus genotype 1b infection and a null response to pegylated interferon-α and ribavirin who developed decompensated graft cirrhosis 6 years after a first liver transplantation. Treatment with sofosbuvir and ribavirin allowed for rapid negativation of serum HCV RNA and was well tolerated despite advanced liver and moderate renal dysfunction. Therapeutic drug monitoring did not reveal any clinically significant drug-drug interactions. Despite virological response, the patient remained severely decompensated and re-transplantation was performed after 46 days of undetectable serum HCV RNA. The patient is doing well 12 months after his second liver transplantation and remains free of hepatitis C virus.

**Conclusions:**

The use of directly acting antivirals may allow for successful liver re-transplantation for recipients who remain decompensated despite virological response and is likely to improve the outcome of liver re-transplantation for end-stage recurrent hepatitis C.

## Background

Chronic hepatitis C (CHC) represents the leading indication to liver transplantation (LT) [1-3]. Recurrent hepatitis C virus (HCV) infection is universal in patients with detectable serum HCV RNA at the time of LT, and 20-40% will develop cirrhosis of the liver graft within 5 years after LT [1-3]. Treatment of recurrent HCV infection has been a challenge, with limited sustained virological response (SVR) rates and often poor tolerability. Hence, recurrent hepatitis C is associated with reduced graft and patient survival [1-3].

Re-transplantation for graft failure due to recurrent hepatitis C is controversial, and some centers abandoned the procedure because of poor outcomes [1,4,5]. HCV RNA in serum would ideally have to be negative before re-transplantation, but this was difficult to achieve until recently.

Outstanding progress has been made in the field of CHC treatment, with the development of potent directly acting antivirals (DAAs) [6,7]. These DAAs are expected to have a major impact on LT for CHC [8,9]. Recently, Curry *et al.* have demonstrated that re-infection of a first liver graft can be prevented by sofosbuvir (SOF; a nucleotide NS5B polymerase inhibitor) and ribavirin (RBV) in patients with compensated cirrhosis awaiting LT for hepatocellular carcinoma due to CHC [10]. Moreover, a seminal case report has shown the successful treatment of fibrosing cholestatic hepatitis with an oral combination of daclatasvir (an NS5A inhibitor) and SOF [11], and studies have demonstrated significantly improved SVR rates in patients treated for recurrent hepatitis C with new DAAs [12,13].

Here, we report the first case to our knowledge of a successful SOF and RBV treatment before liver re-transplantation for terminal graft failure due to recurrent hepatitis C. This treatment regimen allowed for re-transplantation and successfully prevented re-infection of the second liver graft.

## Case presentation

A 54-year-old Caucasian male developed end-stage liver failure due to HCV genotype 1b infection and received LT for a first time in 2007. Recurrent hepatitis C was documented in 2008 (Metavir score A2F1) and treated with pegylated interferon-α (PEG-IFN-α) and RBV, with a null response. Advanced fibrosis (Metavir score A2F3) was documented in 2011. Incipient graft failure and right retinal detachment subsequently precluded IFN-based antiviral therapy. Progressive graft failure was noted toward the end of 2012. At no point there was any evidence of allograft rejection. In September 2013, the Child-Pugh score increased to C10 and model for end-stage liver disease (MELD) score to 26, with jaundice, ascites, edema and profound fatigue. Total bilirubin was 229 μmol/l, albumin 29 g/l, prothrombin time 41%, INR 1.6, and serum creatinine 140 μmol/l. Immunosuppressive treatment consisted of cyclosporine 50 mg bid (with trough levels around 80 μg/l) and prednisone 5 mg qd. Multidisciplinary team discussion concluded to listing of the patient for re-transplantation provided that he could benefit from effective antiviral therapy before his second LT.

**Figure 1:**
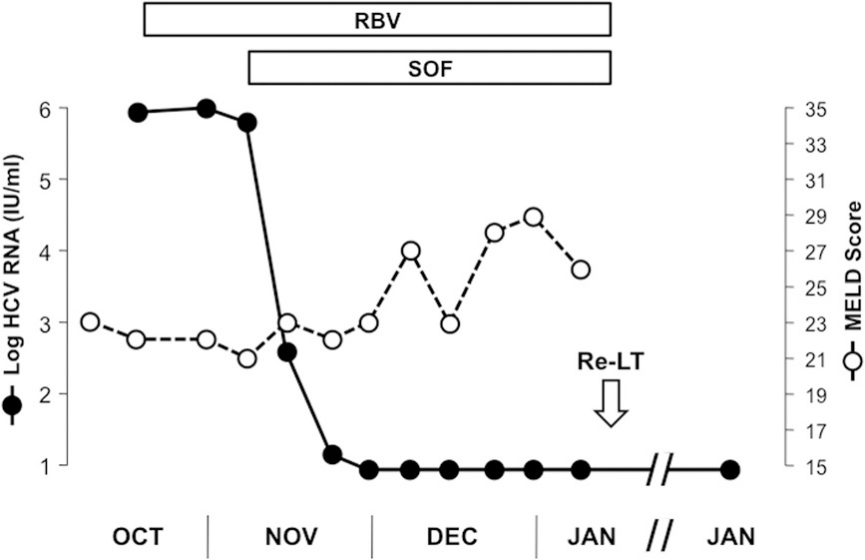
**Evolution of HCV RNA and MELD score in a 54-year-old patient with end-stage recurrent hepatitis C treated with sofosbuvir (SOF) and ribavirin (RBV) prior to liver re-transplantation.** Despite the rapid virological response to SOF and RBV, the recipient remained severely decompensated and liver re-transplantation was performed in January 2014. HCV RNA was undetectable in serum for 46 days prior to liver re-transplantation and remained undetectable on follow-up throughout January 2015 (i.e. more than 1 year post-liver re-transplantation). Re-LT, liver re-transplantation.

SOF was provided by Gilead Sciences Inc. (Foster City, CA) on a compassionate use basis and was started at a dose of 400 mg qd at the beginning of November 2013. Given the impaired renal function, RBV was started 3 weeks prior to SOF to ensure tolerability and pursued thereafter at a daily dose of 400-600 mg. As shown in Figure 1, HCV RNA declined rapidly upon the introduction of SOF and became undetectable 3 weeks later. Treatment was well tolerated and there was no need for the administration of erythropoietin.

The results of therapeutic drug monitoring (TDM) for SOF, RBV and cyclosporine are shown in Table 1. Details of the methodology developed to measure plasma concentrations of SOF, based on liquid chromatography coupled with tandem mass spectrometry, will be reported elsewhere (LAD, unpublished data). RBV plasma concentrations were determined using a previously described analytical method [14]. Cyclosporine trough levels were determined by enzyme multiplied immunoassay on a Cobas Integra® 400 plus system using reagents from Roche Diagnostics (Rotkreuz, Switzerland).

**Table 1:** **Therapeutic drug monitoring and selected laboratory values**

Tx wk	RBV dose (mg)	RBV level (ng/ml)	SOF dose (mg)	SOF level (ng/ml)	CsA dose (mg)	CsA level (μg/l)	Creat. (μmol/l)	INR	Total bilirubin (μmol/l)	HCV RNA (logIU/ml)
**−3**	-	ND	-	-	50-50	79	124	1.5	119	5.9
**−1**	400	873	-	-	50-50	77	121	1.7	102	6.0
**0**	400	1253	-	-	50-50	69	118	1.6	101	5.8
**1**	600	1453	400	ND	50-50	67	132	1.7	102	2.6
**2**	600	2161	400	0.7	50-50	83	113	1.7	118	<1.2 (traces)
**3**	600	2234	400	ND	50-50	91	138	1.7	103	ND
**4**	400	2457	400	0.8	50-50	86	160	1.8	168	ND
**5**	400	1968	400	NA	50-50	103	133	1.8	83	ND
**6**	400	2789	400	1.9	50-25	81	173	2.0	106	ND
**8**	400	2594	400	ND	50-25	63	203	1.9	129	ND
**10**	400	2231	400	ND	50-25	79	188	1.6	116	ND

Treatment weeks (Tx wk) relate to the start of sofosbuvir (SOF). Ribavirin (RBV) was started following the blood drawing on week -3 and SOF following the blood drawing on week 0. Creat., creatinine; CsA, cyclosporine A; INR, international normalized ratio; NA, not available; ND, not detectable; RBV, ribavirin.

As shown in Table 1, SOF was measurable in the ng/ml concentration range in some of the plasma samples obtained 24 hours after drug administration. RBV concentrations reached a steady state between 2000 and 2500 ng/ml after around 4 weeks. Doses of cyclosporine had to be reduced by 25% in the last weeks before re-transplantation likely due to progressive liver dysfunction.

As shown in Figure 1, liver function continued to deteriorate despite the virological response, with an increase of the MELD score to 29. Therefore, re-transplantation was performed in January 2014, after a total of 66 days on SOF and 46 days of undetectable serum HCV RNA. SOF and RBV were both discontinued at re-transplantation.

The postoperative course was uneventful and the patient returned home 4 weeks later. An episode of acute cellular rejection (Banff 7) was treated with high-dose corticosteroid boluses in March 2014. Liver function tests have remained normal in the following and, most importantly, HCV RNA has remained undetectable throughout the entire postoperative course until January 2015, i.e. more than 1 year after re-transplantation.

## Discussion

The management of recurrent HCV infection after LT represented a major challenge until recently. PEG-IFN-α and RBV combination therapy has limited efficacy and is difficult to manage [1-3]. Tolerability remained generally poor and there were significant drug-drug interactions with triple therapy comprising the first-generation NS3-4A protease inhibitors telaprevir or boceprevir [15,16]. However, outcomes have improved greatly with the advent of new DAAs and IFN-free combination therapies [8,9,12,13]. More importantly, these new therapeutic options also offer opportunities to treat HCV infection prior to LT and to prevent re-infection of the graft. In this context, Curry *et al*. recently reported the successful use of SOF and RBV in a series of patients with compensated cirrhosis awaiting a first LT for HCV-related hepatocellular carcinoma [10]. The authors found that HCV RNA negativity in serum for at least 30 days prior to LT prevented HCV re-infection in the majority of patients.

We report here the first case to our knowledge of successful SOF and RBV treatment before re-transplantation of a patient with graft failure due to recurrent hepatitis C, with a previous null response to PEG-IFN-α and RBV combination therapy. Antiviral therapy was well tolerated and resulted in undetectable HCV RNA in serum within 3 weeks. Despite this rapid and complete virological response, liver function continued to deteriorate. This is consistent with a recent report on the use of SOF plus daclatasvir for post-transplant recurrent hepatitis C in which there was a lack of clinical benefit when treatment was given late [17]. Liver re-transplantation was performed after 46 days of undetectable HCV RNA, without re-infection of the second graft.

TDM of SOF, RBV and cyclosporine demonstrated the absence of excessive accumulation of any of these drugs in our patient.

SOF is a prodrug with short half-life (about 0.4 h) and rapid uptake into hepatocytes, followed by phosphorylation to the active triphosphate derivative. While the inactive metabolite GS-331007 accounts for > 90%, SOF itself accounts for only about 4% of the systemic exposure. Hence, the low but still measurable SOF trough plasma concentrations observed at some time points period are consistent with an increase in SOF exposure, expected with impaired liver and renal function as well as the co-administration of cyclosporine. Indeed, an average 143% increase in SOF area under the curve (AUC) was described in patients with severe hepatic impairment [18] and an increase by 61 and 107% was found in mild and moderate renal failure, respectively [19]. In addition, a 4.5-fold increase in SOF AUC was reported in the context of concomitant cyclosporine administration [20]. In our patient, impaired liver function, the administration of cyclosporine and mild to moderate renal impairment likely explain a 6- to 8-fold increase in SOF exposure, consistent with plasma concentrations that are still measurable 24 hours after drug administration. However, no dose adjustment was required in our patient. As reported previously, SOF exposure had no significant impact on cyclosporine levels [20].

## Conclusion

This observation illustrates the successful and safe treatment with SOF and RBV in a patient with decompensated graft failure due to recurrent hepatitis C and the prevention of re-infection after re-transplantation. This regimen was well tolerated even in the setting of liver graft failure with chronic renal insufficiency and immunosuppressive drug therapy. The use of DAAs may allow liver re-transplantation for recipients who remain decompensated despite negativation of serum HCV RNA. These DAAs are likely to improve the outcome of liver re-transplantation for end-stage recurrent hepatitis C.

## Consent

Written informed consent was obtained from the patient for publication of this case report and the accompanying table and figure. A copy of the written consent is available for review by the Editor of this journal.

## Abbreviations

AUC: Area under the curve
CHC: Chronic hepatitis C
DAA: Directly acting antiviral
HCV: Hepatitis C virus
LT: Liver transplantation
MELD: Model for end-stage liver disease
PEG-IFN-α: Pegylated interferon-α
RBV: Ribavirin
SOF: Sofosbuvir
SVR: Sustained virological response
TDM: Therapeutic drug monitoring
